# Assessment of Quarantine Understanding and Adherence to Lockdown Measures During the COVID-19 Pandemic in Palestine: Community Experience and Evidence for Action

**DOI:** 10.3389/fpubh.2021.570242

**Published:** 2021-03-02

**Authors:** Hamzeh Al Zabadi, Noor Yaseen, Thair Alhroub, Maryam Haj-Yahya

**Affiliations:** ^1^Public Health Department, Faculty of Medicine and Health Sciences, An-Najah National University, Nablus, Palestine; ^2^Medicine Department, Faculty of Medicine and Health Sciences, An-Najah National University, Nablus, Palestine

**Keywords:** COVID-19, lockdown, Palestine, pandemic, perceptions, quarantine, adherence

## Abstract

**Background:** Containment of the coronavirus pandemic relied extensively on the combination of early implementation of quarantine and massive behavioral changes to ensure effectiveness. Decision-makers need to constantly monitor the outbreak situation and the impact of the measures implemented. Yet little is known about the factors influencing adherence and understanding of lockdown measures among the Palestinian community. This study aimed to assess the impact and factors affecting these early public health interventions.

**Materials and Methods:** A cross-sectional web-based questionnaire was distributed throughout social media (Facebook and Instagram). We used a snowball recruiting technique to target Palestinian adult citizens during the coronavirus pandemic quarantine between 6 and 16 April 2020, which corresponded to almost the middle interval of the strict massive lockdown measures in Palestine that lasted from 22 March to 5 May 2020. Multivariate logistic regression models were developed for the outcome variables (staying home adherence, in-home precautions adherence, and quarantine understanding).

**Results:** Our questionnaire was completed by 2,819 participants. The mean (range) age was 29.47 (18–71) years. Of them, 1,144 (40.6%), 1,261 (44.7%), and 1,283 (45.5%) reported low levels of staying home adherence, in-home precautions adherence, and quarantine understanding, respectively. Females, city residents, those with higher educational levels, and those informed by official government sources were associated significantly with higher levels of both staying home adherence and quarantine understanding. Adequate food supply was associated with a higher level of staying home adherence. Higher levels of in-home precautions adherence were noticed in the elderly and those with a high-risk group living at home. Higher monthly income was inversely associated with higher levels of in-home precautions adherence and lower levels of quarantine understanding (*P* < 0.05).

**Conclusions:** The socio-economic and financial status of the general population and coordination between the major information resources (official government), social media, and the press were the major factors affecting the community in regard to quarantine adherence. For maximum effectiveness and commitment levels amongst the people to decrease the spread of infection, policymakers need to address all those factors. In addition, clear communication between policymakers and the population is essential for reassuring the people and minimizing their fears regarding the unknown future.

## Background

On 11 March 2020, the WHO declared COVID-19 to be a pandemic (1). As the treatment was mainly symptomatic and supportive, protection, and prevention of infection transmission were the best choices worldwide (2). Quarantine announcements were asserted simultaneously almost all over the world. In Palestine, quarantine took place on 22 March 2020 as the first cases of COVID-19 were confirmed (3).

According to the Center for Disease Control and Prevention (CDC), quarantine was adopted as an obligatory means to separate and restrict the movement of people who had potentially been exposed to a contagious disease. People also had to follow appropriate infection control measures which included bans on large social gatherings, school closures, the ban of weddings, parties, and funerals, closures of entertainment venues, various restrictions on restaurant dining areas and gyms, such as increasing the distance between tables and gym machines and improving ventilation to prevent the virus droplet transmission. Adding to this, travel restrictions and social distancing measures were introduced during quarantine (4–7). These measures were implemented to limit disease spread, morbidities, mortalities, and decrease the burden on the health care system, as witnessed before in history with cholera, plague, and influenza (8).

The utility of quarantine is undetermined, and whether or not overusing it can be of any benefit lacks any scientific basis. However, one thing is certain according to a rapid review on how to improve adherence with quarantine: quarantine does not work if people do not adhere to it (9, 10).

Previous surveys on factors that affect adherence to quarantine in outbreaks were reviewed. Multiple factors were studied to assess their effect on the adherence to quarantine and protective health behaviors such as hand washing, avoiding crowds, and maintaining social distance between individuals. Some of these factors were of direct influence and reflected higher adherence actions, such as knowledge about the infectious disease outbreak and quarantine protocol, the perceived benefits of quarantine, the grasped risks of the disease, and the social norms that pressured others to comply with the quarantine. Other factors were of alternative effects, such as where people got their knowledge of quarantine protocols from, with no difference in adherence rates between those who sourced information from official vs. non-official sources. In addition, practical issues such as financial consequences or employees in insecure jobs who lacked leave entitlement would result in individuals being less likely to comply with social distance measures (11). Trust in the government's public health interventions, pre-existing positive appraisal of the health care system, and trust in the national response predicts more adherence to the quarantine (8, 9, 12).

A study in Norway found that adherence to quarantine has been low, especially after the initial surge of infections faded nationwide, which suggests that people are influenced by the perceived infection risk or that the population experiences quarantine fatigue and a wish to return to normality (12).

Recent studies on the topic of the associated predictors with quarantine and health measure compliance showed that gender, age, geographic area, and employment status, as well as the person's fear for themselves and others to contract COVID-19, were significantly predictive (13).

There is very limited data that evaluates the possible predictors which could influence the general population's staying home adherence and the understanding of quarantine and lockdown measures during the COVID-19 pandemic in Palestine. This study is dedicated to providing a clear vision regarding the situation by expanding on the limited knowledge about the possible implicated factors in quarantine compliance. Overall, this could allow the decision-makers to constantly monitor and maintain the balance between the implementation of quarantine and public health measures.

## Materials and Methods

### Study Population, Sample, and Setting

The target population comprised every Palestinian who lived in the West Bank, Gaza, or Jerusalem during coronavirus-2 quarantine and who was equal to or more than 18 years old. We adopted a cross-sectional web-based survey design to assess the public's adherence to quarantine and infection control instructions during the lockdown of coronavirus-2 pandemic by using an anonymous online questionnaire. Every person had a number that reflected their order by the time they finished the questionnaire. A snowball sampling technique was used and focused on recruiting any Palestinian who lived in Palestine during the pandemic. The online survey was disseminated on Facebook and Instagram to friends and local pages and they were encouraged to pass it on to others. A mandatory question was added on the first page of the questionnaire regarding current residency. Those who reported living outside Palestine were automatically excluded from the study. We were able to recruit 2,819 participants in this study who completely filled and returned the questionnaire, with an age range between 18 and 71 years old.

### Questionnaire Development

After reviewing related factors that affect adherence to quarantine in outbreaks (9, 14, 15), we included additional questions related to the COVID-19 pandemic. The structured questionnaire consisted of questions that covered several areas: (1) informed consent, (2) demographic data, (3) knowledge and concerns about quarantine, and (4) compliance to precautionary measures against coronavirus inside and outside the home. The data collection tool was revised by two experts in the field. Then, a pilot study was performed on 56 volunteers of the author's Facebook friends and relatives and their friends (nine of them were older than 30) for feedback to identify ambiguities, questionnaire structure errors, difficult questions, and to record the time taken to complete the questionnaire. Then we took into consideration their notes and edited them as needed; after that, they reviewed the second version and accepted it.

### Procedure and Ethical Consideration

As the Palestinian Government recommended the public to minimize face-to-face interaction and isolate themselves at home, the questionnaire was distributed electronically. Participants completed it in Arabic through an online survey. Expedited ethical approval was obtained from the Institutional Review Board (IRB) at An-Najah National University. Privacy was strictly protected during the procedure as we avoided any questions that could expose the identity of respondents. Information and the purpose of the study were posted on the first page of the questionnaire. All respondents provided electronic informed consent before starting the questionnaire. Data collection took place over 10 days (6–16 April 2020) which corresponded to almost the middle interval of the massive quarantine in Palestine where restriction measures were at their highest (22 March to 5 May 2020).

### Statistical Analysis

Quarantine understanding outcome reflects the knowledge and information the person has about the pandemic and quarantine regardless of the source. It was initially evaluated through five statements: (1) quarantine is needed where I live, (2) not committing to quarantine will raise the number of cases, (3) measures taken by the government are necessary, (4) quarantine should not only be limited to infected people and those who are in contact with them, and (5) hygiene measures in the house are part of quarantine. A 5-point Likert scale [strongly agree (4), agree (3), neutral (2), disagree (1), and totally disagree (0)] was used to respond to each statement. By summing the points of each statement, a scale from 0 to 20 was created for each respondent. We then used the median as a cutoff point to categorize this outcome into a low level (0–17) and a high level (18–20).

Staying home adherence outcome reflects the compliance of the individual to the main instruction given by the government: “Do not leave the house if it is not necessary.” It was initially evaluated through five statements: (1) going grocery shopping or to the bakery, (2) going out meeting friends or family, (3) going out to spend time and have fun, (4) attending social events, and (5) going to the pharmacy. The answer to each statement is composed of [never going out (3), some days (2), more than half of days (1), and every day (0)].

In-home precautions adherence outcome reflects the compliance to infection control measures while staying inside the home to decrease the spread of infection between family members. It was initially evaluated through five statements: (1) washing your hands for 20 seconds or more, (2) decrease the time of interaction with other family members, (3) washing hands after returning from outside, (4) sneezing appropriately according to guidelines (using a tissue or using elbow), and (5) not sharing towels and items between family members. The answer to each statement is composed of [never do them (0), do them sometimes (1), do them most of the time (2), and always do them (3)].

For these last two outcomes separately, we summed up the points of each statement. A scale from 0 to 15 was created for each respondent. Then the median was used as the cutoff point to categorize staying home adherence outcome to a low level (0–12) and a high level (13–15) while categorizing in-home precautions adherence outcome to a low level (0–10) and a high level (11–15).

The 27th version of IBM SPSS (IBM SPSS Statistics for Windows, Version 27.0. Armonk, NY: IBM Corp) was used for data coding, entry, and analysis. All parts of the analysis were performed by the authors themselves. Descriptive statistics (median, mean, standard deviation, and independent student *t*-test) were calculated for continuous variables while frequencies/percentages and Chi-square test were used for categorical variables. *P* < 0.05 was always considered significant.

Statistically significant variables in bivariate analysis were included in the multivariate logistic regression model developed for each of the study outcomes.

## Results

### Socio-Demographic Characteristics of the Study Population

In this study, the questionnaire was introduced to 2,819 individuals, all of whom completed and returned the questionnaire electronically (Table 1). The mean (range) age of respondents was 29.47 (18–71) years. We divided the population into three groups according to age: young (18–35), middle (36–53), and elderly (>53). 73.9% were young and only 4% of participants were elderly. More than two-thirds (72.6%) of respondents were female and nearly half (51.4%) were single. The majority live in the West Bank (83.5%). Most of the participants (78.4%) currently study in college or had graduated recently. Almost one-quarter (24.6%) were smokers. Only 11.8% were health care workers and 45.5% admitted that they had a high-risk group living with them currently.

**Table 1:** Bivariate analysis of socio-demographic characteristics with dependent variables (Staying home adherence; In-home precautions adherence; Quarantine understanding; *P*-value presented was Chi-square significance; *N* = 2,819).

**Variables**	***N* (%)**	**Staying home** **adherence**		***P-*value**	**In-home precautions** **adherence**		***P-*value**	**Quarantine** **understanding**		***P-*value**
**Age**		**Low level**	**High level**		**Low level**	**High level**		**Low level**	**High level**	
		***n* = 1,144**	***n* = 1,675**		***n* = 1,261**	***n* = 1,558**		***n* = 1,283**	***n* = 1,536**	
18–35	2,083(73.9)	825(39.6)	1,258(60.4)	0.106	979(47)	1,104(53)	<0.001^*^	897(43.1)	1,186(56.9)	<0.001^*^
36–53	624(22.1)	276(44.2)	348(55.8)		247(39.6)	377(60.4)		329(52.7)	295(47.3)	
>53	112(4)	43(38.4)	69(61.6)		35(31.3)	77(68.7)		57(50.9)	55(49.1)	
**Sex**										
Male	768(27.2)	468(60.9)	300(39.1)	<0.001^*^	377(49.1)	391(50.9)	0.04^*^	409(53.3)	359(46.7)	<0.001^*^
Female	2,051(72.8)	676(33)	1,375(67)		884(43.1)	1,167(56.9)		874(42.6)	1,177(57.4)	
**Social status**										
Single	1,449(51.4)	539(37.2)	910(62.8)	<0.001^*^	669(46.2)	780(53.8)	0.114	593(40.9)	856(59.1)	<0.001^*^
Relationship	1,370(48.6)	605(44.2)	765(55.8)		592(43.2)	778(56.8)		690(50.4)	680(49.6)	
**Residency**										
Village	1,380(49)	618(44.8)	762(55.2)	<0.001^*^	631(45.7)	749(54.3)	0.113	657(47.6)	723(52.4)	0.01^*^
City	1,292(45.8)	463(35.8)	829(64.2)		576(44.6)	716(55.4)		550(42.6)	742(57.4)	
Camp	147(5.2)	63(42.9)	84(57.1)		54(36.7)	93(63.3)		76(51.7)	71(48.3)	
**Geographic area**										
West bank	2,354(83.5)	969(41.6)	1,385(58.4)	0.03^*^	1,060(45)	1,294(55)	0.768	1,059(45)	1,295(55)	0.014^*^
Gaza strip	270(9.6)	118(43.7)	152(56.3)		116(43)	154(57)		144(53.3)	126(46.7)	
Jerusalem	195(6.9)	57(29.2)	138(71.8)		85(43.6)	110(56.4)		80(41)	115(59)	
**Educational level**										
Secondary or less	326(11.6)	166(50.9)	160(49.1)	<0.001^*^	151(46.3)	175(53.7)	0.068	207(63.5)	119(36.5)	<0.001^*^
Collage	2,211(78.4)	865(39.1)	1,346(60.9)		1,002(45.3)	1,209(54.7)		964(43.6)	1,247(56.4)	
Master or doctorate	282(10)	113(40.1)	169(59.9)		108(38.3)	174(61.7)		112(39.7)	170(60.3)	
**Health care worker**										
Yes	332(11.8)	131(39.5)	201(60.5)	0.657	139(41.9)	193(58.1)	0.264	141(42.5)	191(57.5)	0.236
No	2,487(88.2)	1,013(40.7)	1,474(59.3)		1,122(45.1)	1,365(54.9)		1,142(45.9)	1,345(54.1)	
**Monthly income (Shekel)**										
<2,000	568(20.1)	240(42.3)	328(57.7)	0.512	232(40.9)	336(59.)	0.032^*^	297(52.3)	271(47.7)	<0.001^*^
2,000–5,000	1,552(55.1)	631(40.7)	921(59.3)		692(44.6)	860(55.4)		706(45.5)	846(54.5)	
>5,000	699(24.8)	273(39.1)	426(60.9)		337(48.2)	362(51.8)		280(40.1)	419(59.9)	
**Smoking/Shisha**										
Yes	693(24.6)	350(50.5)	343(49.5)	<0.001^*^	328(47.3)	365(52.7)	0.113	363(52.4)	330(47.6)	<0.001^*^
No	2,126(75.4)	794(37.4)	1,332(62.6)		933(43.9)	1,193(56.1)		920(43.3)	1,266(56.7)	
**High risk group in home**										
Yes	1,283(45.5)	539(42)	744(58)	0.158	536(41.8)	747(58.2)	0.004^*^	705(55)	831(45)	0.653
No	1,536(54.5)	605(39.4)	931(60.6)		725(47.2)	811(52.8)		578(37.6)	705(62.4)	

* *P-value is statistically significant*.

It was found that 1,144 (40.6%), 1,261 (44.7%), and 1,283 (45.5%) of respondents had low levels of staying home adherence, in-home precautions adherence, and quarantine understanding, respectively.

### Quarantine Characteristics of the Population

As shown in Table 2 98% of respondents believed that quarantine is important, and 2,173 (77.1%) expressed fear of getting COVID-19 or transmitting it to others. Only 14.9% of respondents had jobs that required them to leave home during quarantine, and only 85 (3%) had at least one of their relatives infected with COVID-19. The two most common sources of information about quarantine and precautions were social media and television/radio (59.5% and 18.6%, respectively). Nearly 80.2% admitted that they were properly informed about the quarantine, and 29.3% documented inadequate food supplies to withstand the quarantine period.

**Table 2:** Bivariate analysis of quarantine characteristics with dependent variables (Staying home adherence; In-home precautions adherence; Quarantine understanding; *P*-value presented was Chi-square significance; *N* = 2,819).

**Variables**	***N* (%)**	**Staying home adherence**			**In-home precautions adherence**			**Quarantine understanding**		
		**Low level**	**High level**	***P-*value**	**Low level**	**High level**	***P-*value**	**Low level**	**High level**	***P-*value**
		***n* = 1,144**	***n* = 1,675**		***n* = 1,261**	***n* = 1,558**		***n* = 1,283**	***n* = 1,536**	
**Do you think quarantine is important?**										
Yes	2,763(98)	1,116(40.4)	1,647(59.6)	0.147	1,232(44.6)	1,531(55.4)	0.248	1,238(44.8)	1,525(55.2)	<0.001^*^
No	56(2)	28(50)	28(50)		29(51.8)	27(48.2)		45(80.4)	11(19.6)	
**Type of quarantine**										
Obliged to stay at home	2,398(85.1)	902(37.6)	1,496(62.4)	<0.001^*^	1,046(43.6)	1,334(56.4)	0.356	1,060(44.2)	1,338(55.8)	0.001^*^
I have to work outside home	421(14.9)	242(57.5)	179(42.5)		197(46.8)	224(35.2)		223(53)	198(47)	
**Any of relatives or acquainted infected?**										
Yes	85(3)	34(40)	51(60)	0.912	40(47.1)	45(52.9)	0.661	39(45.9)	46(54.1)	0.945
No	2,734(97)	1,110(40.6)	1,624(59.4)		1,221(44.7)	1,513(55.3)		1,244(45.5)	1,490(54.5)	
**Afraid of getting COVID-19 or transmit it?**										
Yes	2,173(77.1)	852(39.2)	1,321(60.8)	0.006^*^	950(43.7)	1,223(56.3)	0.047^*^	897(41.3)	1,276(58.7)	<0.001^*^
No	646(22.9)	292(45.2)	354(54.8)		311(48.1)	335(51.9)		386(59.8)	260(40.2)	
**Properly informed about quarantine**										
Yes	2,262(80.2)	884(39.1)	1,378(60.9)	0.001^*^	976(43.2)	1,286(56.8)	0.001^*^	984(43.5)	1,278(56.5)	<0.001^*^
No	557(19.8)	260(46.7)	279(53.3)		285(51.2)	272(48.8)		299(53.7)	258(46.3)	
**Source of information**										
Television or radio	525(18.6)	219(41.7)	306(58.3)	0.027^*^	221(42.1)	304(57.9)	<0.001^*^	259(49.3)	266(50.7)	<0.001^*^
Official government agencies	359(12.7)	134(37.3)	225(62.7)		120(33.4)	239(66.6)		132(36.8)	227(63.2)	
A health care worker	159(5.6)	67(42.1)	92(57.9)		58(36.5)	101(63.5)		63(39.6)	96(60.4)	
Social media	1,676(59.5)	669(39.9)	1,007(60.1)		806(48.1)	870(51.9)		770(45.9)	906(54.1)	
Conversation with other people	100(3.6)	55(55)	45(45)		56(56)	44(44)		59(59)	41(41)	
**Enough food supply to withstand quarantine period?**										
Yes	1,994(70.7)	750(37.6)	1,244(62.4)	<0.001^*^	876(43.9)	1,118(56.1)	0.184	855(42.9)	1,139(57.1)	<0.001^*^
No	825(29.3)	394(47.8)	431(52.2)		385(46.7)	440(53.3)		428(51.9)	397(48.1)	
**Quarantine duration**										
1–2 weeks	187(6.6)	98(52.4)	89(47.6)	<0.001^*^	86(46)	101(54)	0.103	102(54.5)	85(45.5)	0.023^*^
2–3 weeks	847(30.1)	355(41.9)	847(58.1)		357(42.2)	490(57.8)		396(46.8)	847(53.2)	
3–4 weeks	786(27.9)	331(42.1)	786(57.9)		378(48.1)	408(51.9)		357(45.4)	786(54.6)	
>4 weeks	999(35.4)	360(36)	639(67)		440(44)	559(56)		428(42.8)	571(57.2)	
**Average hours out home before quarantine**										
<2 h	584(20.7)	206(35.3)	378(64.7)	<0.001^*^	261(44.7)	323(55.3)	0.851	289(49.5)	295(50.5)	0.020^*^
2–6 h	776(27.5)	337(43.4)	439(56.6)		356(45.9)	776(54.1)		344(44.3)	432(55.7)	
6–10 h	1,075(38.2)	415(38.6)	660(61.4)		478(44.5)	1,075(55.5)		460(42.8)	615(57.2)	
>10 h	384(13.6)	186(48.4)	198(51.6)		166(43.2)	384(56.8)		190(49.5)	194(50.5)	

* *P-value is statistically significant*.

However, most people (38.2%) used to spend between 6 and 10 h outside the home before the quarantine. Most respondents (94.1%) correctly identified that quarantine aimed to protect society. Only 52.6% understood that quarantine restrictions aimed to protect members of their household. Nearly 59.4% correctly reported that quarantine would not protect them.

### Bivariate Analysis of the Study Main Outcomes

Staying home adherence outcome was found to have statistically significant associations with the following socio-demographic variables [(sex, social status, residency, geographic area, educational level, and smoking); *P* < 0.05, Table 1] and the following quarantine characteristic variables [(quarantine type, fear of getting COVID-19 or transmitting it, being properly informed about quarantine, source of information, having an adequate food supply, quarantine duration, and average hours outside the home before quarantine); *P* < 0.05, Table 2].

Regarding in-home precautions adherence outcome, statistically significant associations were found with the following socio-demographics [(age, sex, monthly income, and high-risk group in the home); *P* < 0.05, Table 1] and the following quarantine characteristics [(fear of getting COVID-19 or transmitting it, being properly informed about quarantine, and source of information); *P* < 0.05, Table 2].

On the other side, quarantine understanding outcome was found to be significantly associated with these socio-demographics [(age, sex, social status, residency, geographic area, educational level, monthly income, and smoking); *P* < 0.05, Table 1] and the following quarantine characteristic variables [(belief in the importance of quarantine, quarantine type, fear of getting COVID-19 or transmitting it, being properly informed about quarantine, source of information, having enough food supply, quarantine duration, and average hours outside the home before quarantine); *P* < 0.05, Table 2].

**Figure 1:**
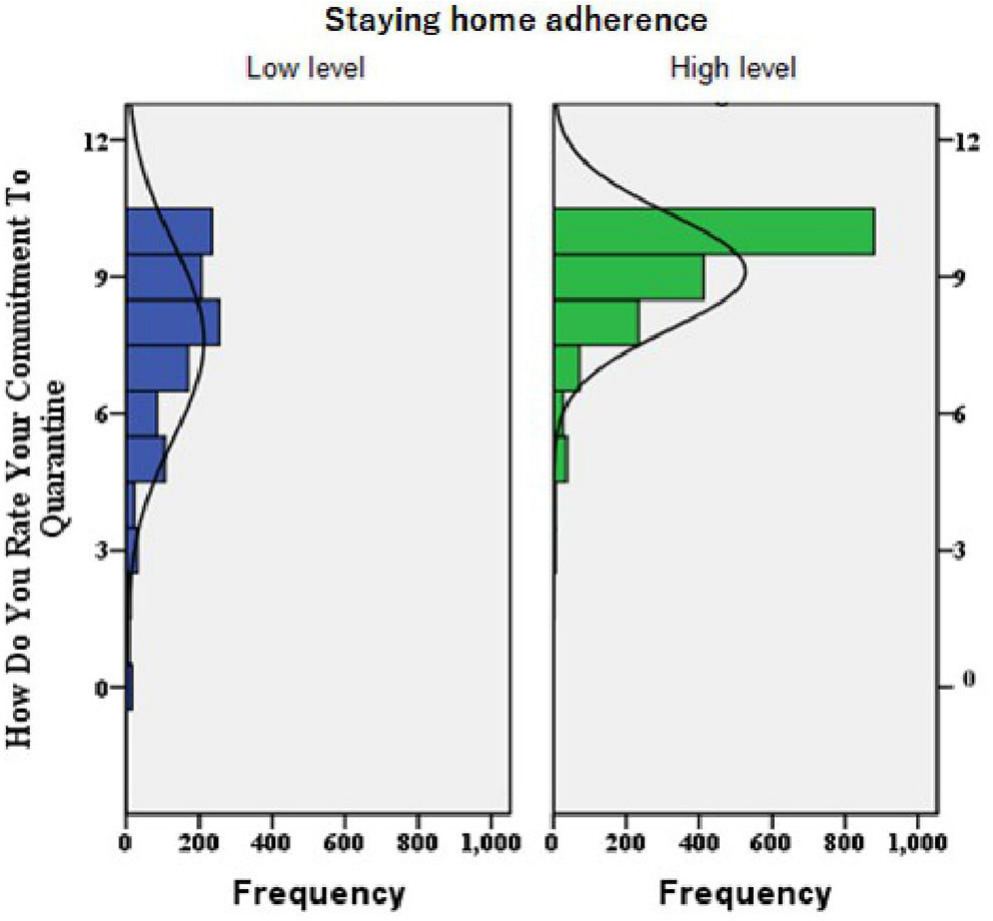
A histogram chart built for the distribution of participants' self-rating of adherence to quarantine among the high and low levels of staying home adherence (*P*-value of *t*-test analysis for the mean difference between the two groups was <0.001; i.e., High-level group showed significantly higher Mean ± SD self-rating of adherence to quarantine compared to low-level, 9.11 ± 1.26 and 7.56 ± 2.15, respectively).

As shown in Figure 1, those with higher mean scores of the self-reported rating of adherence to quarantine were significantly more likely to have a high-level of staying home adherence compared to those who reported lower mean scores (mean ± SD = 9.11 ± 1.26 and 7.56 ± 2.15; *P* < 0.001; respectively).

### Multivariate Logistic Regression for Staying Home Adherence

The multivariate logistic regression model for staying home adherence outcome predictors is shown in Table 3. As shown, female sex, city residents, and higher educational levels (master and doctorate) were associated with a higher level of staying home adherence [ORs (95%CIs) = 2.72 (2.27–3.37); 1.37 (1.16–1.64); and 1.51 (1.06–2.16); respectively]. Furthermore, being informed through official government sources and having adequate food supply were more likely to result in a higher level of staying home adherence [ORs (95%CIs) = 1.38 (1.03–1.86) and 1.23 (1.03–1.47); respectively). Being obliged to stay at home was also a significant positive predictor of a higher level of staying home adherence. On the contrary, being in a relationship (engaged or married) was inversely related to staying home adherence [OR (95%CI) = 0.7 (0.59–0.83)]. Other variables did not remain significant after adjusting for other variables in the model.

**Table 3:** Multivariate logistic regression model for factors associated with staying home adherence (*N* = 2,819).

**Explanatory variable**	**Beta coefficient**	**Standard error**	**AOR^**!**^**	**95% CI^**~**^**	***P*-value**
**Sex**					
Female	1.02	0.10	2.77	2.27–3.37	<0.001
Male^*^	–	–	–	–	–
**Social status**					
Relationship	−0.36	0.09	0.70	0.59–0.83	<0.001
Single^*^	–	–	–	–	–
**Residency**					
City	0.32	0.09	1.37	1.16–1.64	<0.001
Camp	0.27	0.19	1.31	0.90–1.92	0.162
Village^*^	–	–	–	–	–
**Geographic area**					
West bank	−0.34	0.17	0.71	0.51–1.0	0.053
Gaza	−0.63	0.22	0.54	0.35–0.82	0.004
Jerusalem^*^	–	–	–	–	–
**Educational level**					
Collage	0.24	0.13	1.27	0.98–1.64	0.070
Master or doctorate	0.41	0.18	1.51	1.06–2.16	0.023
Secondary or less^*^	–	–	–	–	–
**Smoking/Shisha**					
Yes	−0.12	0.10	0.89	0.73–1.09	0.246
No^*^	–	–	–	–	–
**Type of quarantine**					
I am obliged to stay at home	0.63	0.12	1.87	1.49–2.34	<0.001
My work requires that I stay outdoors^*^	–	–	–	–	–
**Afraid of getting COVID-19 or transmit it?**					
Yes	0.18	0.10	1.2	0.99–1.45	0.063
No^*^	–	–	–	–	–
**Do you think that you have been properly informed about quarantine?**					
Yes	0.18	0.10	1.19	0.98–1.46	0.087
No^*^	–	–	–	–	–
**Source of information**					
Official government agencies	0.32	0.15	1.38	1.03–1.86	0.031
A health care worker	0.02	0.20	0.02	0.70–1.51	0.903
Social media	0.09	0.11	1.09	0.89–1.35	0.405
Conversations with other people	−0.31	0.24	0.74	0.46–1.17	0.193
Television or radio^*^	–	–	–	–	–
**Enough food supply to withstand quarantine period**					
Yes	0.21	0.09	1.23	1.03–1.47	0.023
No^*^	–	–	–	–	–
**Quarantine duration**					
2–3 Weeks	0.23	0.17	1.26	0.90–1.77	0.186
3–4 Weeks	0.15	0.18	1.16	0.82–1.63	0.407
>4 Weeks	0.30	0.17	1.35	0.97–1.90	0.080
1–2 Weeks^*^	–	–	–	–	–
**Average hours out of home before quarantine**					
<2 h	0.27	0.15	1.32	0.99–1.76	0.063
2–6 h	−0.13	0.14	0.88	0.67–1.15	0.338
6–10 h	0.11	0.13	1.11	0.86–1.44	0.421
>10 h^*^	–	–	–	–	–

* *Reference category. ^~^CI, Confidence interval; ^!^AOR, Adjusted odds ratio (AOR for high level as compared with low level). Enter method was used*.

### Multivariate Logistic Regression for In-Home Precautions Adherence

In-home precautions adherence model shown in Table 4; older age groups (36–53 and 54–71 years) showed strong positive associations with a higher level of in-home precautions adherence compared to younger adults [OR (95%CI) = 1.37 (1.14–1.66) and 2.17 (1.42–3.30); respectively].

**Table 4:** Multivariate logistic regression model for factors associated with in-home precautions adherence (*N* = 2,819).

**Explanatory variable**	**Beta coefficient**	**Standard error**	**AOR^**!**^**	**95% CI^**~**^**	***P*-value**
**Sex**					
Female	0.30	0.09	1.35	1.14–1.61	0.001
Male^*^	–	–	–	–	–
**Age**					
36–53	0.32	0.10	1.37	1.14–1.66	0.001
54–71	0.77	0.22	2.17	1.42–3.30	<0.001
18–35^*^	–	–	–	–	–
**Monthly income (shekel)**					
2,000–5,000	−0.19	0.10	0.83	0.68–1.01	0.066
>5,000	−0.33	0.12	0.72	0.57–0.90	0.005
<2,000^*^	–	–	–	–	–
**High risk group in home**					
Yes	0.21	0.08	1.23	1.06–1.43	0.008
No^*^	–	–	–	–	–
**Afraid of getting COVID-19 or transmit it?**					
Yes	0.14	0.09	1.15	0.96–1.38	0.120
No^*^	–	–	–	–	–
**Do you think that you have been properly informed about quarantine?**					
Yes	0.21	0.10	1.23	1.01–1.49	0.036
No^*^	–	–	–	–	–
**Source of information**					
Official government agencies	0.46	0.15	1.58	1.19–2.10	0.002
A health care worker	0.33	0.19	1.40	0.96–2.03	0.080
Social media	−0.19	0.10	0.83	0.68–1.02	0.070
Conversations with other people	−0.36	0.23	0.70	0.45–1.09	0.113
Television or radio^*^	–	–	–	–	–

* *Reference category. ^~^CI, Confidence interval; ^!^AOR, Adjusted odds ratio (AOR for high level as compared with low level). Enter method was used*.

Furthermore, female sex, having a high-risk group in the home, and considering official government agencies as a source of information were significantly associated with a higher level of in-home precautions adherence [OR (95%CI) = 1.35 (1.14–1.61), 1.23 (1.06–1.43), and 1.58 (1.19–2.10); respectively]. Being properly informed about quarantine was also a significant positive predictor. On the other hand, higher monthly income (>5,000 Shekels) was inversely related to in-home precautions adherence [OR (95%CI) = 0.72 (0.57–0.90)].

### Multivariate Logistic Regression for Quarantine Understanding

The multivariate logistic regression model for quarantine understanding outcome predictors is shown in Table 5. Female sex, city residents, and a higher educational level (master and doctorate) were associated with a higher level of quarantine understanding [ORs (95%CIs) = 1.29 (1.06–1.58); 1.21 (1.02–1.44); and 2.29 (1.60–3.27); respectively]. Furthermore, being informed through official government sources, being properly informed, and fear of catching COVID-19 were significant predictors of a higher level of quarantine understanding [ORs (95%CIs) = 1.64 (1.23–2.20); 1.32 (1.08–1.62); and 2.03 (1.68–2.45); respectively]. Moreover, higher monthly income (>5,000 shekels), being obligated to stay at home, and those who believe that quarantine is important were associated with a higher level of quarantine understanding. On the contrary, being in a relationship (engaged or married) and smokers (cigarette or shisha) were inversely related to quarantine understanding [OR (95%CI) = 0.71 (0.59–0.85) and 0.80 (0.66–0.97); respectively]. Other variables did not remain significant after adjusting for other variables in the model.

**Table 5:** Multivariate logistic regression model for factors associated with quarantine understanding (*N* = 2,819).

**Explanatory variable**	**Beta coefficient**	**Standard error**	**AOR^**!**^**	**95% CI^**~**^**	***P*-value**
**Sex**					
Female	0.26	0.10	1.29	1.06–1.58	0.012
Male^*^	–	–	–	–	–
**Age**					
36–53	−0.30	0.11	0.74	0.60–0.93	0.008
54–71	−0.05	0.22	0.95	0.62–1.45	0.817
18–35^*^	–	–	–	–	–
**Social status**					
Relationship	−0.35	0.10	0.71	0.59–0.85	<0.001
Single^*^	–	–	–	–	–
**Residency**					
City	0.19	0.09	1.21	1.02–1.44	0.025
Camp	−0.00	0.19	0.10	0.69–1.45	0.984
Village^*^	–	–	–	–	–
**Geographic area**					
West bank	0.03	0.16	1.03	0.75–1.42	0.858
Gaza	−0.45	0.21	0.64	0.42–0.97	0.035
Jerusalem^*^	–	–	–	–	–
**Educational level**					
Collage	0.53	0.13	1.69	1.30–2.19	<0.001
Master or doctorate	0.83	0.18	2.29	1.60–3.27	<0.001
Secondary or less^*^	–	–	–	–	–
**Monthly income (shekel)**					
2,000–5,000	0.16	0.11	1.17	0.95–1.45	0.146
>5,000	0.27	0.13	1.31	1.01–1.69	0.041
<2,000^*^	–	–	–	–	–
**Smoking/Shisha**					
Yes	−0.23	0.10	0.80	0.66–0.97	0.025
No^*^	–	–	–	–	–
**Do you think quarantine is important?**					
Yes	1.28	0.36	3.61	1.79–7.25	<0.001
No^*^	–	–	–	–	–
**Type of quarantine**					
I am obliged to stay at home	0.28	0.11	1.33	1.06–1.66	0.012
My work requires that I stay outdoors^*^	–	–	–	–	–
**Afraid of getting COVID-19 or transmit it?**					
Yes	0.71	0.10	2.03	1.68–2.45	<0.001
No^*^	–	–	–	–	–
**Do you think that you have been properly informed about quarantine?**					
Yes	0.28	0.10	1.32	1.08–1.62	0.007
No^*^	–	–	–	–	–
**Source of information**					
Official government agencies	0.50	0.15	1.64	1.23–2.20	0.001
A health care worker	0.36	0.20	1.44	0.98–2.11	0.061
Social media	0.12	0.11	1.12	0.91–1.38	0.276
Conversations with other people	−0.30	0.24	0.74	0.46–1.17	0.200
Television or radio^*^	–	–	–	–	–
**Enough food supply to withstand quarantine period**					
Yes	0.09	0.09	1.10	0.92–1.32	0.309
No^*^	–	–	–	–	–
**Quarantine duration**					
2–3 Weeks	0.19	0.17	1.20	0.86–1.68	0.279
3–4 Weeks	0.20	0.17	1.22	0.87–1.71	0.240
>4 Weeks	0.23	0.17	1.26	0.91–1.76	0.169
1–2 Weeks^*^	–	–	–	–	–
**Average hours out of home before quarantine**					
<2 h	−0.04	0.14	0.96	0.73–1.28	0.785
2–6 h	0.10	0.14	1.10	0.84–1.44	0.488
6–10 h	0.11	0.13	1.12	0.87–1.44	0.378
>10 h^*^	–	–	–	–	–

* *Reference category. ^~^CI, Confidence interval; ^!^AOR, Adjusted odds ratio (AOR for high level as compared with low level). Enter method was used*.

## Discussion

The present study aimed to assess staying home adherence, in-home precautions adherence, and quarantine understanding among Palestinian society during the COVID-19 pandemic lockdown.

Females, city residents, those with a higher level of education, those obliged to stay at home as a type of quarantine, and those considering official government agencies as a source of information were associated with a higher level of staying home adherence and quarantine understanding. This could be explained by the cultural background of Palestinian society where males usually spend more time working outside the home. In our study, 47% of females and 64.4% of males reported more than 6 h on average outside the home before the quarantine. Police forces are usually more distributed in city centers compared to villages, and cities are usually more crowded; therefore the risk of COVID-19 is higher. On one hand, higher educated-people understand the risk of transmission and infection more which could affect their understanding and adherence compared to less-educated individuals. On the other hand, higher-educated people usually have jobs that can be performed from the home through online applications, whereas less-educated people usually have craft jobs that require them to leave the home. In a study in Israel during the same pandemic, it was noted that the compliance rate to self-isolation was affected by loss of income, as the compliance rate dropped from 94 to 57% when income was not compensated through the government (16). A study during the H1N1 pandemic quarantine in Victoria found that people who used official sources of information only compared to those who used both official and unofficial sources showed no differences in the odds of compliance (Odds Ratio 1.00, 95% CI = 0.69–1.44) (17). Official sources of information are usually trusted and considered as a clear source that people commit to and understand more clearly. However, in Australia, a study during the H1N1 pandemic found no differences in adherence rates between those who took the information from official vs. nonofficial sources (9), whereas in a Canadian study, the source of information was found to be significantly associated with quarantine understanding (18).

Having an adequate food supply in the home was associated with a higher level of staying home adherence. It is reasonable that those who secure their food resources before the quarantine can avoid leaving the home easily in contrast to others who will be worried about protect their family from starving. However, monthly income (>5,000 Shekels), fear of getting COVID-19 or transmitting it, and being properly informed about quarantine were associated with a high-level of quarantine understanding. These again reinforce the importance of proper delivery of information to the public and the underlying fears from COVID-19 transmission and infection rate. An Australian study during the H1N1 pandemic reported that people who understand quarantine were more compliant with it compared to people who reported inadequacy of information (17). However, in the UK, a study during the COVID-19 pandemic (16) reported that functional fear rather than sociopolitical factors increased compliance rates, which highlights the effect of fear on public response. Those with higher monthly incomes might not have resisted the quarantine and they understood it more as they had enough currency and didn't worry about financial shortages. It is noteworthy that loss of income was found to be the most frequently cited problem in compliance with quarantine (19).

However, being in a relationship (engaged or married) was inversely related to a higher level of both staying home adherence and quarantine understanding. This may be in part due to more responsibilities toward household members to supply the home with what is needed during the quarantine. On the other side, anxiety and stress might play a role in this due to over-stress between family members during the quarantine. Therefore, going out could be an opportunity to relax and to avoid more stress. Smoking (cigarette or Shisha) was inversely associated with quarantine understanding. Cigarette/Shisha smokers usually seek meeting friends more than nonsmokers. They are usually stressed and might not be able to handle and understand quarantine intentionally due to their carelessness and under-estimation of the risk. Moreover, the effect of financial status on their ability of smoking due to job loss may make them more stressed.

The elderly and those with a high-risk group living with them were more likely to have higher in-home precautions adherence. It is worth mentioning that the elderly are usually considered a high-risk group if infected with COVID-19, and by the time of the study, the only two deaths from COVID-19 in Palestine were two elderly patients with co-morbidities (20). In-home precautions adherence might be considered crucial to protect those with a high-risk group in the home. Conversely, during a mumps outbreak at an American University, isolation compliance didn't significantly differ by gender, age, location of residence, or employment status (21). Surprisingly, high monthly income (>5,000 Shekels) was inversely associated with in-home precautions adherence. This is in opposition to our expectations as those with a higher monthly income can usually afford to buy sanitizers and protective equipment. But it seems that the ability to buy differs from adherence. Their feelings of being able to be treated if infected might affect their adherence as they thought they have more currency for better affordable treatment. Furthermore, those people might have a higher nutritional status and were not afraid of COVID-19 infection and thought that they were strong enough not to catch the infection, mainly due to false information during COVID-19. A more likely explanation is that people with a monthly income of more than 5,000 Shekels have a healthier family, and are less likely to have a high-risk group in the home. In our study, 42.2% of people who had >5,000 Shekels as monthly income reported having a high-risk group in the home, while 57.8% of people with monthly income <5,000 Shekels reported having a high-risk group in the home.

It should be noted that only two factors (females and those who consider official government agencies as a source of information) were significantly associated with a higher level of the three study outcomes. Average hours spent outside of the home before quarantine and duration of quarantine did not affect any of the study outcomes. This is in accordance with other studies during the H1N1 pandemic in Australia (17). People might appreciate more factors and the severity of the disease and its transmission for adherence and quarantine understanding than the length of quarantine and the hours they usually spent outside of the home before the quarantine. Forcing the quarantine through the declaration of emergency bylaws might leave people to concentrate more on staying at home rather than the length of quarantine itself. As the study focused on factors affecting adherence to quarantine measures and one of the inclusion criteria was Palestinian individuals in Palestine under quarantine, the West Bank had the highest number of responders because both Gaza and Jerusalem were not under lockdown until the last few days of the study.

This study could have some limitations. Selection bias could have occurred due to the sampling technique. Due to social distancing during quarantine, we disseminated the survey on social media, and this might in part exclude people who do not have access to the internet and social media, and also limit access to children and the elderly. Any participant who was younger than 18-years-old was excluded. Furthermore, only 4% of the participants were older than 53 years old. However, according to Index Mundi, only 8% of the Palestinian population were older than 55 years old, and around 36% of the population were younger than 15 years in 2020 (22), We believe that the elderly use the internet less frequently than other age groups, and for this reason, although the elderly group had been represented to some extent in our sample (4%), our study could not represent all age groups. However, this was the only possible procedure to perform during the lockdown measures and it was useful in collecting the required information as fast and safely as possible. Systematic bias where over- or under-estimation of some measures due to self-reporting might also have been encountered. This study has several strengths, including a large sample size and the sampling timeframe that corresponded to the peak surge of COVID-19 cases in Palestine, which has had 613 cases and five deaths as per writing this paper (20). From an epidemiological point of view, our study might not represent the national level; however, taking into account the worldwide nature of the risk in this pandemic, we strongly believe that these data could provide useful information to be generalized to other countries and future pandemics.

## Conclusions

It was seen that major effects depend mainly on the socio-economic and financial status of the general population and the coordination between the major information resources (official government), social media, and the press. Hence, addressing such factors could enable the country to achieve higher adherence rates that can effectively decrease the spread of infection. It is important for policymakers to reach out to the community by every possible means during the lockdown to prevent the spread of false news, enhance their understanding, and update them with new measures. Policymakers' clear communication with the people is crucial for their reassurance, as such communication minimizes their fears of the unknown future. As financial status has a great role in the level of adherence, compensation of income loss and giving access to online jobs may decrease the burden of these lockdown measures on the population and ensure higher compliance.

## Data Availability Statement

The raw data supporting the conclusions of this article will be made available by the authors, without undue reservation.

## Ethics Statement

The studies involving human participants were reviewed and approved by IRB of An-Najah National University. The ethics committee waived the requirement of written informed consent for participation.

## Author Contributions

HA, NY, TA, and MH-Y designed study protocol and drafting the manuscript. HA coordinated the study protocol and conducted the statistical analysis. NY, TA, and MH-Y collected the data. All authors read and approved the final manuscript.

## Conflict of Interest

The authors declare that the research was conducted in the absence of any commercial or financial relationships that could be construed as a potential conflict of interest. The reviewer MA declared a shared affiliation with the authors to the handling editor at time of review.
